# Corneal Asphericity and Higher-Order Aberrations after FS-LASIK and Trans-PRK for Myopia

**DOI:** 10.1155/2021/3765046

**Published:** 2021-12-06

**Authors:** Yuan Wu, Shuhan Wang, Guiqin Wang, Shaozhen Zhao, Ruihua Wei, Yue Huang

**Affiliations:** ^1^Tianjin Key Laboratory of Retinal Functions and Diseases, Tianjin 300384, China; ^2^Tianjin Branch of National Clinical Research Center for Ocular Disease, Tianjin 300384, China; ^3^Eye Institute and School of Optometry, Tianjin Medical University Eye Hospital, Tianjin 300384, China

## Abstract

**Objective:**

To compare the corneal asphericity and higher-order aberrations (HOAs) of femtosecond laser-assisted in situ keratomileusis (FS-LASIK) with Smart Pulse Technology (SPT) assisted transepithelial photorefractive keratectomy (Trans-PRK) for myopia and myopic astigmatism correction.

**Methods:**

This prospective study analyzed 88 eyes of 44 patients treated with FS-LASIK and 64 eyes of 32 patients treated with Trans-PRK. All eyes had low to moderate myopia with or without astigmatism (spherical equivalent (SE) <−6.00 diopters). The uncorrected distance visual acuity (UDVA), corrected distance visual acuity (CDVA), SE, asphericity (*Q* value) of the anterior corneal surface, index of surface variance (ISV), corneal higher-order aberrations (HOAs), vertical coma (Z_3_^−1^), horizontal coma (Z_3_^1^), and spherical aberration (*Z*_4_^0^) over a 6 mm diameter central corneal zone diameter were evaluated preoperatively and 1, 3, and 6 months postoperatively.

**Results:**

At 6 months, the UDVA and SE were −0.14 ± 0.06 and 0.33 ± 0.33D in FS-LASIK and −0.15 ± 0.06 and 0.35 ± 0.37D in Trans-PRK. There was no difference between the two groups in the postoperative UDVA and SE (*P* > 0.05). After FS-LASIK and Trans-PRK, the *Q* values in the 6, 7, 8, and 9 mm zones and ISV of the anterior corneal surface significantly increased (*P* < 0.001). At 1, 3, and 6 months after surgery, corneal HOA, Z_3_^−1^, *Z*_3_^1^, and *Z*_4_^0^ in both groups were significantly increased compared with those before surgery, with statistically significant differences (*P* < 0.001). At 1, 3, and 6 months after surgery, the Z_3_^−1^ of the Trans-PRK group was significantly lower than that of the FS-LASIK group (*P* < 0.001). ΔHOA and ΔZ_4_^0^ were dramatically correlated with the ΔQ value for both FS-LASIK and Trans-PRK procedures. The ΔQ was significantly correlated with the preoperative SE, AD, and AD/CCT after both two procedures (all *P* < 0.001).

**Conclusions:**

Both FS-LASIK and Trans-PRK caused the anterior corneal surface to become flatter, and the morphology of the corneal surface was irregular. Corneal HOAs were significantly increased after the two procedures. Trans-PRK using SPT introduced less corneal vertical coma than FS-LASIK. Corneal asphericity changes contributed to the corneal aberrations changes following FS-LASIK and Trans-PRK.

## 1. Introduction

Corneal refractive surgery has developed rapidly, and its safety, efficacy, and predictability have been continuously improved with the development of science and technology. Femtosecond laser-assisted in situ keratomileusis (FS-LASIK) and small incision lenticule extraction (SMILE) are widely used in clinical practice due to their accuracy in making corneal flap and small incision, respectively. Transepithelial photorefractive keratectomy (Trans-PRK) has become a highly researched topic in recent years. It is characterized by one-step removal of the corneal epithelium and correction of refractive errors, which shortens the operation time and avoids the complications caused by FS-LASIK to make the corneal flap [1]. With this breakthrough, Smart Pulse Technology (SPT) has been applied to excimer laser to smooth the corneal surfaces, resulting in faster postoperative recovery from Trans-PRK [2].

In principle, corneal refractive surgery aims to correct refractive errors by ablating a certain amount of corneal stroma in the central optical zone of the cornea. More central stromal tissue is ablated than peripheral tissue, which inevitably causes flattening of the cornea and causes changes in the shape of the anterior corneal surface inevitably. Many studies have shown that the corneal asphericity developed significantly from negative value to positive value before and after surgery [3–5], and the corneal morphology became more flatter. There is a certain correlation between the changes in corneal morphology and the introduction of higher-order aberrations (HOAs) [6, 7]. In addition, HOAs are thought to be related to poor night vision, glare, halo, and visual quality [8], affecting patient satisfaction and long-term curative effects.

Previous studies have found that the morphological changes of the anterior corneal surface after SMILE and FS-LASIK are different [9], and the introduction of HOAs into SMILE after surgery is lower than that into FS-LASIK. Zhang et al. [10] showed that Trans-PRK changes the corneal curvature to a greater extent and the visual quality (*Q*-value, aberrations) to a lesser extent than FS-LASIK. This study was conducted to compare the change patterns of corneal aberrations and corneal asphericity between FS-LASIK and Trans-PRK using SPT and to investigate how changes of corneal asphericity could account for the variation of corneal aberrations after the two procedures.

## 2. Patients and Methods

### 2.1. Patients and Study Design

This prospective study included patients with low to moderate myopia, with or without astigmatism, who presented to the Tianjin Medical University Eye Hospital, Tianjin, China consecutivelyfrom July 2019 to January 2020. A nonrandomized controlled study design was used. Patients were divided into two groups: the case group for whom Trans-PRK was performed and the control group who received FS-LASIK. There were 44 patients (88 eyes) in the FS-LASIK group, including 23 males and 21 females. In the Trans-PRK group, there were 32 patients (64 eyes), including 15 males and 17 females. Inclusion criteria were as follows: age over 18 years with stable refraction for at least 2 years (the annual increase of myopic diopter was no more than 0.50 D); discontinuation of soft contact lens wear for a minimum of 2 weeks and rigid contact lens wear for at least 1 month prior to preoperative examination; myopia ≤−6.00D and astigmatism ≤ −2.00D; and no keratoconus tendency, no active eye disease, or systemic disease. Exclusion criteria were as follows: abnormal or keratoconic topography, active inflammation in the eyes, presence of periocular purulence, presence of serious ocular appendage lesions, history of previous ocular surgery, and presence of concurrent ocular diseases and systemic diseases that could affect corneal wound healing. The choice of surgical procedure primarily depended on the patients' preference (after a detailed description of the procedures). All patients signed informed consent.

### 2.2. Ocular Examination

All patients underwent routine preoperative complete eye examinations including measurement of the uncorrected (UDVA) and corrected (CDVA) distance visual acuity, manifest and cycloplegic refraction, slit-lamp evaluation of the anterior and posterior segment, intraocular pressure (IOP), axial length, keratometry, and corneal topography (Pentacam; Oculus, Germany). The *Q* value and corneal aberration were measured using the Pentacam in a dark room. The patient was positioned and instructed to focus on automatic measurement immediately after blinking to obtain data so as to avoid interference caused by poor tear film quality and eyelid occlusion of the patient. The imaging quality result was shown as OK, and the corneal exposed area was greater than 9 mm. The *Q* values at diameters of 6, 7, 8, and 9 mm were measured on the anterior surface of the cornea. Corneal higher-order aberrations (HOAs), vertical coma (Z_3_^−1^), horizontal coma (*Z*_3_^1^), and spherical aberration (*Z*_4_^0^) were measured in a diameter range of 6 mm.

### 2.3. Surgical Procedures

FS-LASIK and Trans-PRK were performed by the same skilled surgeon.

In the FS-LASIK group, levofloxacin and 0.3% sodium hyaluronate eye drops were administered for 3 days before surgery, and 0.4% obuvacaine hydrochloride eye drops were used for surface anesthesia. The cornea flap was created with the IntraLase FS femtosecond laser system (USA). The flap hinge was located in the upper cornea. The flap diameter was set at 8.5 mm. According to the patient's own corneal thickness and ablation depth, the thickness of the corneal flap was set at a range of 95–110 *μ*m. After all the bubbles under the corneal flap were absorbed and the flap was lifted, refractive ablation was performed with the Amaris 1050 excimer laser (Schwind, eye-tech-solutions, Germany). The ORK-CAM software was used for surgical design, and the standard aspheric aberration-free mode was used. The surgeon asks the patient to look at the correct position during the ablation process. After the ablation, the eye was washed with a balanced salt solution and the corneal flap was repositioned. All patients were prescribed levofloxacin eye drops qid for 3 days, 0.1% fluoromethane drops qid for 4 weeks (reducing later to once a week), and 0.3% sodium hyaluronate drops qid for 3 months.

In the Trans-PRK group, levofloxacin and 0.3% sodium hyaluronate eye drops qid for 3 days were administered before surgery, prognac eye drops were administered 3 times (once every 15 min) before surgery to reduce the inflammatory response and pain, and 0.4% obuvacaine hydrochloride eye drops were used for surface anesthesia. All surgeries were performed with the Amaris 1050 excimer laser (Schwind eye-tech-solutions, Germany). The ORK-CAM software is used for surgical design. The epithelial thickness was set to 55 *μ*m in the center and 65 *μ*m in the periphery in the 8 mm diameter range. The standard aspheric aberration-free mode of Trans-PRK using SPT is used for laser ablation of epithelium and stroma. Patients were requested to look at a pulsing green fixation light throughout the ablation. After laser ablation, the ocular surface was then thoroughly washed with cooled balanced salt solution, a bandage contact lens (Pure Vision, Bausch & Lomb) was applied, and 0.3% tobramycin dexamethasone eye drops were instilled. All patients were instructed to use a topical instillation of levofloxacin, 0.1% fluoromethane, and sodium bromophenolate hydrate eye drops qid until removal of the contact lens. Following the healing of the corneal epithelium, we prescribed 0.1% fluoromethane drops qid for the first month (then reducing to once a month) and 0.3% sodium hyaluronate drops qid for 4 months.

### 2.4. Statistical Analysis

Data were analyzed using IBM SPSS version 26.0 (IBM Inc., New York, USA). The normality of the data was verified with the Kolmogorov–Smirnov test. The independent sample *t* test was used to compare the general data between the two groups. Δ*Q*, ΔHOA, ΔZ_3_^−1^, ΔZ_3_^1^, and ΔZ_4_^0^, respectively, represent the difference between each measurement value 6 months after operation and before operation. All corneal aberration results were statistically calculated using the root mean square (RMS) value. A repeated measurement two-way analysis of variance (ANOVA) was used to analyze the overall variation of parameters. The least-significant difference (LSD) was used for pairwise comparisons between and within groups. Pearson's correlation and simple regression were used to analyze the correlation. *P* values less than 0.05 were considered statistically significant.

## 3. Results

There were no significant differences in any of the variables between the FS-LASIK and Trans-PRK groups (*P* > 0 05). The detailed baseline characteristics by group are provided in Table 1.

### 3.1. Corneal Asphericity

**Figure 1:**
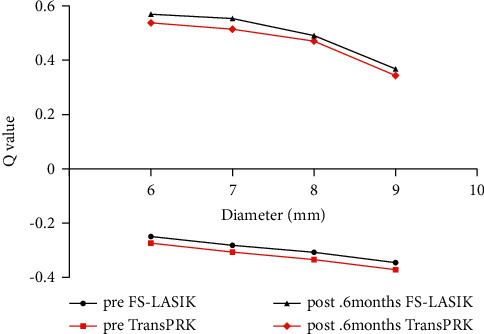
Comparison of the *Q* value changes in the FS-LASIK and Trans-PRK groups. The *Q* value of the anterior corneal surface significantly shifted to a positive value after the FS-LASIK and Trans-PRK groups. No significant difference between FS-LASIK and Trans-PRK in the changes of the *Q* value of the anterior corneal surface was found.

The *Q* value before surgery was negative for the anterior corneal surface in both groups. After FS-LASIK and Trans-PRK, the *Q* value in the 6, 7, 8, and 9 mm zones of the anterior corneal surface significantly shifted to a positive value (*P* < 0.001) (Table 2, Figure 1). Compared with the FS-LASIK group, the Q values of each diameter range of the Trans-PRK group were smaller at 1, 3, and 6 months after surgery, but there was no statistically significant difference between the two groups (*P* > 0.05) (Table 2).

**Figure 2:**
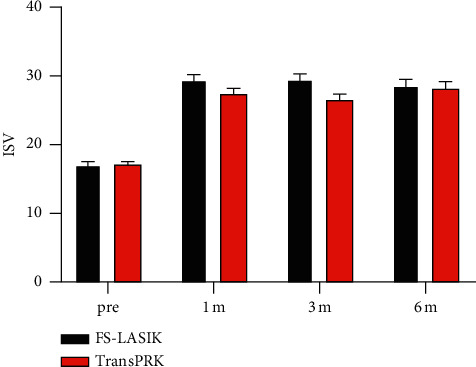
Preoperative and postoperative ISV values in the FS-LASIK and Trans-PRK groups. The ISV of the corneal surface significantly increased after the FS-LASIK and Trans-PRK groups. No significant difference between FS-LASIK and Trans-PRK in the changes of the ISV was found. ISV, index of surface variance.

The postoperative ISV value of the cornea in both groups was significantly increased compared with that before surgery. After surgery, ISV values were significantly different from those before surgery (*F*_time_ = 218.324, *P* < 0.001). There was no significant difference in preoperative and postoperative ISV values between the two groups (*F*_group_ = 2.012, *P*=0.159) (Figure 2).

### 3.2. Corneal Aberrations

**Figure 3:**
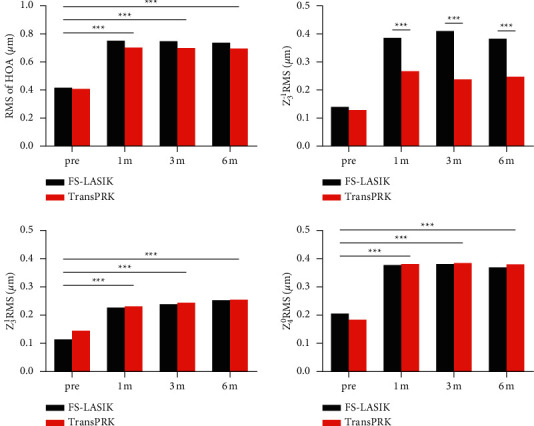
Comparison of the corneal higher-order aberrations (HOAs), vertical coma (*Z*_3_^−1^), horizontal coma (*Z*_3_^1^), and spherical aberration (*Z*_4_^0^) between FS-LASIK and Trans-PRK surgery. No significant differences for the corneal HOAs, *Z*_3_^1^, and *Z*_4_^0^ between the two groups were found. The *Z*_3_^−1^ in the Trans-PRK group was significantly lower compared with the FS-LASIK group. ^*∗∗∗*^*P* < 0.001.

The changes in corneal HOAs, Z_3_^−1^, *Z*_3_^1^, and *Z*_4_^0^ in both groups after surgery as compared to the preoperative data are shown in Figure 3. The root mean square (RMS) in HOAs, Z_3_^−1^, *Z*_3_^1^, and *Z*_4_^0^ for the both groups increased after surgery (all *P* < 0.001). There were no significant differences for the corneal HOAs, *Z*_3_^1^, and *Z*_4_^0^ between the two groups (*P* > 0.05). Only the Z_3_^−1^ in the Trans-PRK group was significantly lower than that of the FS-LASIK group (*P* < 0.001). Regarding the variation in the aberration, only Δ Z_3_^−1^ showed statistically significant difference between the two groups (*P* < 0.05), while Δ HOA, Δ *Z*_3_^1^, and Δ *Z*_4_^0^ showed no statistically significant differences between the two groups (*P* > 0.05) (Table 3).

### 3.3. Correlation and Regression Analysis

**Figure 4:**
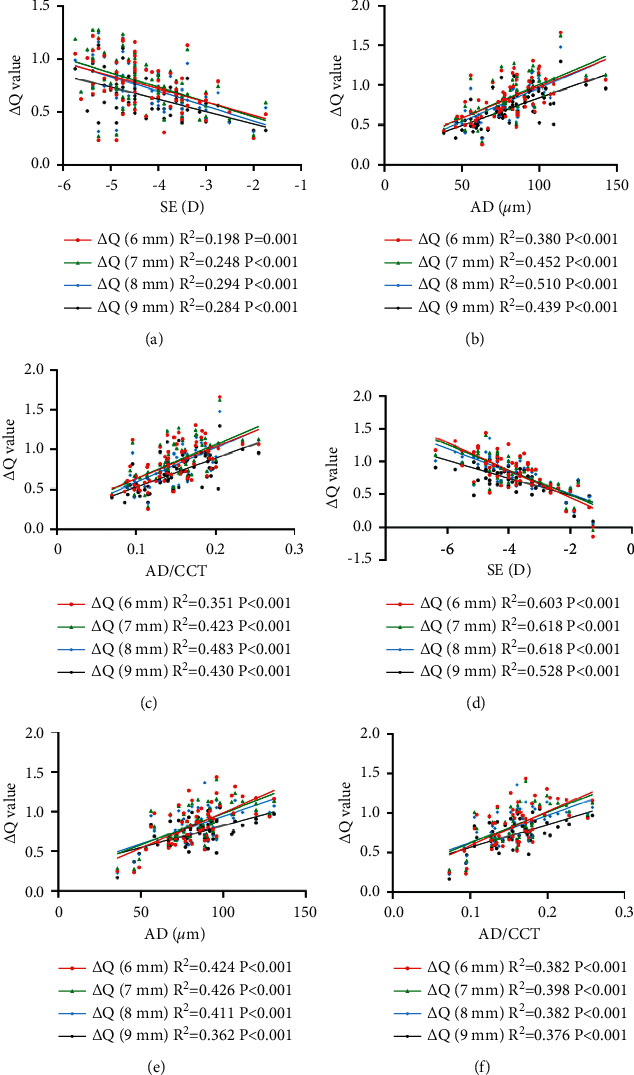
Correlation between the change in *Q* value and spherical equivalent (SE), ablation depth (AD), and the ratio of AD and central corneal thickness (AD/CCT) after the FS-LASIK (a–c) and Trans-PRK (d–f) procedures.

**Figure 5:**
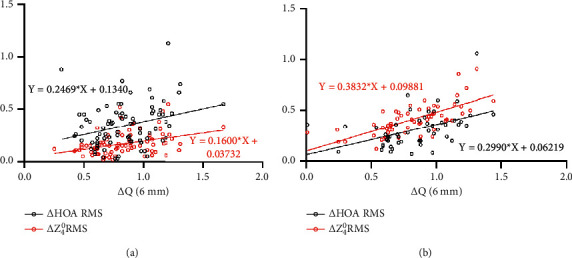
Correlation between the change of *Q* value and the change of HOA and spherical aberration (*Z*_4_^0^) in the FS-LASIK (a) and Trans-PRK (b) procedures.

Data for the correlation analysis performed by group postoperatively are shown in Figures 4 and 5. The Δ*Q* value was significantly correlated with the preoperative spherical equivalent (SE), ablation depth (AD), and the ratio of AD and CCT (AD/CCT) after both the FS-LASIK and Trans-PRK procedures (all *P* < 0.001) (Figure 4). The Δ HOA and Δ *Z*_4_^0^ were positively correlated with the Δ*Q* value at the anterior corneal surface within 6 mm diameter after both the two procedures (FS-LASIK: *r* = 0.303, *P*=0.005; *r* = 0.370, *P* < 0.001; Trans-PRK: *r* = 0.467, *P* < 0.001; *r* = 0.695, *P* < 0.001, respectively) (Figure 5).

## 4. Discussion

In clinics, FS-LASIK and Trans-PRK are widely used in the correction of myopia. The safety and effectiveness of these two procedures have been widely verified [11–13]. However, both procedures inevitably changed the asphericity of the cornea and introduced higher-order aberrations (HOAs).

The anterior corneal surface flattens gradually from the center to the periphery in physiological conditions. The corneal asphericity (*Q* value) at the anterior corneal surface was negative and increased from the center to the periphery. Our results showed that the *Q* value of the anterior corneal surface changed from negative to positive after FS-LASIK and Trans-PRK in the 6, 7, 8, and 9 mm zones. There was no statistically significant difference between the two groups. Nonetheless, we found that *Q* values after Trans-PRK surgery were lower than those of FS-LASIK at 1, 3, and 6 months. This may be the advantage of the Trans-PRK procedure. In addition, our results showed that ISV significantly increased after FS-LASIK and Trans-PRK. Although there was no significant difference between the two procedures, the value of ISV was lower at each follow-up time after Trans-PRK than that of FS-LASIK, which further indicated that Trans-PRK had certain advantages over FS-LASIK in maintaining the corneal surface regularity and retaining the morphology of the anterior surface. Regarding the changes of corneal morphology after surgery, the following reasons should be considered: First, the corneal epithelium undergoes a repair process after corneal refractive surgery, which can make the anterior surface of the cornea smooth. This is manifested as an uneven thickening of the corneal epithelium [14], that is, the degree of thickening of the center is inconsistent with that of the periphery [15]. This may be the main cause of postoperative corneal morphological changes. Second, the energy and angle transferred to the center and periphery of the cornea may be different during laser ablation. This may result in the corneal morphology to be altered. Since the Amaris 1050 laser was used during the stroma ablation in the two procedures in this study, there were no statistically significant differences in the changes of corneal morphology between the two groups. The slight clinical differences between the two groups may be related to postoperative corneal biomechanics, corneal nerve recovery, and healing response differences.

The main sources of ocular aberrations are the cornea and lens including corneal aberrations and intraocular aberrations. Corneal refractive surgery was performed on the surface of the cornea, and the morphology of the anterior corneal surface changed significantly after surgery without affecting the lens. Therefore, corneal aberration was selected in the current study to evaluate the aberrations after refractive surgery. Zheng et al. [16] previously indicated that significant HOAs induction is common after refractive surgery, and the creation of corneal flap, changes in corneal biomechanical properties after surgery, and corneal wound healing may be the reasons for the increase of HOAs [17]. Our results showed that corneal HOAs, corneal coma, and spherical aberration were significantly increased after the two procedures, which indicated that although the surgery eliminated low-order aberrations such as myopia and astigmatism and improved uncorrected distance visual acuity, the two procedures increased corneal HOAs. The increase in coma is thought to be related to the creation of the corneal flap and to minor intraoperative eye movements. FS-LASIK has a corneal flap incision and the flap hinge is located at the 12 o'clock position of the cornea, while Trans-PRK surgery has no incision, thus the integrity of the cornea is better maintained. This may be the main reason why the vertical coma in the FS-LASIK was significantly higher than that in the Trans-PRK. A previous study [18] showed that a significant horizontal coma is introduced if the corneal flap hinge is created on the nasal side. In addition, despite the use of a 7-dimensional eye-tracking system in both procedures, the increase of coma caused by eccentric ablation could not be completely avoided due to the uncontrollable factors of patients during the operation. The increase in spherical aberration is mainly related to the changes of corneal asphericity caused by corneal epithelial healing and matrix fibrosis. Postoperative corneal thinning and biomechanical changes also have a certain impact on corneal asphericity and HOAs. Adib-Moghaddam et al. [12] followed up the clinical results 18 months after Trans-PRK and found that corneal spherical aberration and coma were significantly increased after the surgery. Feng et al. [19] found that corneal coma, spherical aberration, and total HOAs were significantly increased at 1 week, 1 month, and 3 months after the surgery compared with the preoperative values. These results were consistent with our report. A recent study [20] followed up for 12 months to compare the changes of corneal HOAs between Trans-PRK and FS-LASIK for high myopia, and found that corneal HOAs and vertical coma in the Trans-PRK group were statistically significantly lower than those in the FS-LASIK group, while the horizontal coma and spherical aberration of the cornea were similar between the two groups. We believe that diopter difference may be the reason for the difference between the results of this study and those of theirs.

Another finding of our study was that the changes in corneal HOAs and spherical aberration were dramatically correlated with the increase in *Q* value for both FS-LASIK and Trans-PRK procedures. We also found that the increase in *Q* value was significantly correlated with the preoperative SE, AD, and AD/CCT after the two procedures. Zheng et al. [16] believed that the changes of corneal HOAs were mainly caused by the morphological changes of the cornea after surgery. In addition, many other factors may affect the results, such as the stability of laser energy during surgery, instability of the tear film, and corneal healing. Previous studies [18] have shown that the asphericity of the prolate cornea and the asphericity of the oblate lens are mutually compensated to obtain a smaller spherical aberration and a better visual quality. After corneal refractive surgery, the corneal asphericity is significantly altered. This morphological change causes the light through the peripherial cornea to focus earlier than the central cornea, thus showing an increase in postoperative corneal aberrations. The higher the myopic degree is, the deeper the corneal stroma that needs ablation is, and the transition from the central area to the periphery of the cornea becomes steeper, which may cause a large change in *Q* value, thus leading to an increase in HOAs of the cornea, especially spherical aberrations. Roberts and Dupps et al. [21, 22] propose that central ablation can reduce the tension in residual peripheral lamellar segments, leading to an outward peripheral force to pull the center toward the periphery, resulting in central corneal flattening after myopic ablation. Considering that this outward pull destroys the corneal morphology, it further causes changes in corneal aberrations.

## 5. Conclusions

In summary, our study found that both FS-LASIK and Trans-PRK caused the anterior corneal surface to become flatter, and the morphology of the corneal surface to become irregular. The corneal higher-order aberrations were significantly increased after the two procedures. Trans-PRK using SPT introduced less corneal vertical coma than FS-LASIK. Corneal asphericity changes contributed to the corneal aberration changes following FS-LASIK and Trans-PRK. This should be considered when selecting patients for refractive surgery.

## Figures and Tables

**Table 1:** Preoperative characteristics by group.

Parameters	FS-LASIK (*n* = 44)	Trans-PRK (*n* = 32)	*P* value
Age (years)	25.32 ± 6.00	26.60 ± 6.96	0.40
UDVA (logMAR)	1.04 ± 0.28	1.03 ± 0.32	0.79
CDVA (logMAR)	0.01 ± 0.02	0.00 ± 0.03	0.09
Sphere (D)	−3.92 ± 0.95	−3.89 ± 1.05	0.87
Cylinder (D)	−0.75 ± 0.41	−0.71 ± 0.36	0.62
SE (D)	−4.27 ± 1.02	−4.06 ± 1.09	0.34
CCT (*μ*m)	540.49 ± 29.60	532.83 ± 30.28	0.13
K1 (D)	42.98 ± 1.32	42.89 ± 1.09	0.64
K2 (D)	44.05 ± 1.34	43.93 ± 1.20	0.56

FS-LASIK: femtosecond laser-assisted in situ keratomileusis; Trans-PRK: transepithelial photorefractive keratectomy; CDVA: corrected distance visual acuity (with spectacles); UDVA: uncorrected distance visual acuity; SE: spherical equivalent; D: diopters, CCT: central corneal thickness; K1: cornea flat meridian curvature; K2: cornea steep meridian curvature.

**Table 2:** Asphericity (*Q* value) of the anterior corneal surface in FS-LASIK and Trans-PRK.

Dia. (mm)	Groups	Preoperative	1 month	3 months	6 months	*P* value (group)	*P* value (time)
6	FS-LASIK	−0.24 ± 0.07	0.61 ± 0.26	0.59 ± 0.25	0.56 ± 0.26	0.324	<0.001
	Trans-PRK	−0.27 ± 0.08	0.56 ± 0.30	0.53 ± 0.31	0.54 ± 0.30		

7	FS-LASIK	−0.28 ± 0.08	0.61 ± 0.25	0.57 ± 0.24	0.55 ± 0.25	0.249	<0.001
	Trans-PRK	−0.30 ± 0.08	0.54 ± 0.28	0.51 ± 0.29	0.51 ± 0.28		

8	FS-LASIK	−0.31 ± 0.09	0.55 ± 0.23	0.50 ± 0.23	0.48 ± 0.24	0.207	<0.001
	Trans-PRK	−0.33 ± 0.09	0.48 ± 0.26	0.45 ± 0.26	0.46 ± 0.26		

9	FS-LASIK	−0.35 ± 0.10	0.41 ± 0.22	0.38 ± 0.21	0.36 ± 0.22	0.381	<0.001
	Trans-PRK	−0.37 ± 0.10	0.36 ± 0.24	0.35 ± 0.23	0.34 ± 0.24		

FS-LASIK: femtosecond laser-assisted in situ keratomileusis; Trans-PRK: transepithelial photorefractive keratectomy; Dia: diameter.

**Table 3:** Comparison of the corneal aberrations between FS-LASIK and Trans-PRK (mean ± SD, *μ*m).

Groups	HOA	Vertical coma (*Z*_3_^−1^)	Horizontal coma (*Z*_3_^1^)	Spherical aberration (*Z*_4_^0^)
Pre-FS-LASIK	0.42 ± 0.07	0.14 ± 0.10	0.11 ± 0.07	0.20 ± 0.06
Pre-Trans-PRK	0.41 ± 0.09	0.13 ± 0.11	0.14 ± 0.09	0.18 ± 0.07
*P* value	0.763	0.505	0.063	0.087
Post-FS-LASIK	0.73 ± 0.14	0.38 ± 0.17	0.25 ± 0.17	0.37 ± 0.11
Post-Trans-PRK	0.72 ± 0.19	0.24 ± 0.15	0.26 ± 0.18	0.38 ± 0.15
*P* value	0.830	0.001^*∗*^	0.801	0.080
Δ FS-LASIK	0.31 ± 0.25	0.24 ± 0.18	0.14 ± 0.16	0.17 ± 0.11
Δ Trans-PRK	0.31 ± 0.18	0.11 ± 0.15	0.12 ± 0.18	0.20 ± 0.15
*P* value	0.530	0.006^*∗*^	0.785	0.063

HOA: higher-order aberration; SD: standard deviation; pre: preoperative, post: 6-month postoperative; Δ FS-LASIK = post-FS-LASIK – pre-FS-LASIK; Δ Trans-PRK = post-Trans-PRK – pre-Trans-PRK. ^*∗*^*P* < 0.05.

## Data Availability

The data used and analyzed in the current study are available from the corresponding author upon reasonable request.
